# Study of Matrix Metalloproteinase-2 in Inguinal Hernia

**DOI:** 10.4021/jocmr2009.12.1281

**Published:** 2009-12-28

**Authors:** Vinod Jain, Rohit Srivastava, Shweta Jha, Samir Misra, Nagendra Singh Rawat, Devendra Vijai Amla

**Affiliations:** a Department of General Surgery, Chhatrapati Shahuji Maharaj Medical University, Lucknow, India; b National Botanical Research Institute, Lucknow, India

## Abstract

**Background:**

Abnormal collagen metabolism is thought to play an important role in the development of primary inguinal and ventral hernia. The detection of an impaired collagen balance both in the tissue as well as in cultured fibroblasts underlines the suspicion that the development of hernia is likely to be implemented primarily by a disturbance of the fibroblast function and their collagen genes. Based on these results we assume that the altered collagen synthesis in hernia patients can be regarded as a genetically linked deregulation serving as a basic initiating or promoting factor for the development of primary inguinal hernias. With the hypothesis that hernia is a local manifestation of a systemic disease manifested by increased expression of matrix metallo-proteinase-2 (MMP-2), a study was planned with following aims: 1) to establish a causal association between inguinal hernia and MMP-2; 2) to test the hypothesis that hernia is a local manifestation of a systemic disorder rather than a mere local mechanical defect.

**Methods:**

A case control study was conducted on 30 subjects of each direct and indirect inguinal hernia and 30 controls. DAC-ELISA test was used for analysis of serum (preoperative) and tissue samples (fascia transversalis) in patients as well as controls.

**Results:**

Statistically, serum levels of MMP-2 were significantly increased in all the hernia patients as compared to controls. This increment was maximum in patients of direct hernia. MMP-2 was not detectable in tissue samples.

**Conclusions:**

Hernia is a local manifestation of a systemic disease rather than a mere local mechanical defect.

**Keywords:**

MMP-2; Matrix Metalloproteinase-2; Inguinal hernia; DAC-ELISA; Collagen metabolism; PBST-Phosphate Buffer Saline Tween-20

## Introduction

Usually an abdominal wall hernia is regarded as a mechanical problem with a local defect which has to be closed technically, either by sutures or, in modern time, with meshes. In the long history of hernia repair, even the most experienced surgeon, irrespective of the utilized technique, has to face recurrences that have been treated by him and correspondingly have to be regarded as his personal technical failure. That is why it is obviously impossible to make mechanical repair with similar success rates in hernia surgery as for engineering [1,2].

The close causal relationship between one technical component and its failure is reflected by s-shaped survival curve. If the recurrence is considered just as a technical failure, it should occur either soon or with a certain delay, but in any case the outcome curve should reveal an s-shaped configuration. However, this contradicts the actual proportions. On the contrary, in incisional and inguinal hernia formation, the cumulative incidences show a linear rise over years without any s-shaped deformation [3,4]. This course is in contradiction to any significant direct causal relationship between technique and recurrence. Instead, an underlying multifactor process has to be suggested. Furthermore, because most of the recurrences occur after 1 year within the linear rise of the cumulative incidences, a multifactor process seems to be far more important than any accusable factor of the early postoperative course.

There is a close association between inguinal hernia and collagen metabolism. A decreased collagen types I/III ratio is found in adult patients with groin hernia as well as in the scar of patients with recurrent hernia [5,6]. Collagen type I is characteristic for mature scars or fascial tissue while the collagen type III represents the mechanically instable, less cross-linked collagen synthesized during the early days of wound healing. Correspondingly, in patients with recurrent hernias, there seems to be an impaired maturation of scar tissue which is not able to close the hernia gap or fix the mesh in place for long. Consequently, a recurrence may develop either through a scar or at the border of a synthetic mesh through its scary fixation.

Abnormal collagen metabolism is thought to play an important role in the development of primary inguinal hernia. This view is strengthened by detection of altered collagen metabolism and structural changes of the tissue in these patients. Several connective tissue diseases have been related to an abnormal collagen metabolism. Patients with aortic abdominal aneurysm [7,8], Ehlers-Danlos Syndrome [9], or Polycystic Kidney Disease [10] show an increased risk for inguinal herniation. Furthermore, previous studies on protein level indicate that patients with an inguinal hernia present a disturbed collagen proportion with a reduced ratio of type I and type III collagen as well as abnormal ultra-structural changes of the deposited collagen [11,12]. A defective collagen metabolism contributes to a decreased tensile strength and mechanical stability of both the connective tissues and the induced scar tissue. Therefore, these alterations in collagen formation should be of central relevance in the pathophysiology of hernias.

The altered ratio of the collagen subtypes can result either by a modified synthesis or by an imbalanced breakdown. The cleavage is regulated by the activity of the matrix metallo-proteinases (MMPs), proteins of a family of zinc-dependent endopeptidases. Among them, matrix metallo-proteinase-1 (MMP-1) and MMP-13 are the principal matrix enzymes cleaving fibrillar type I, II and III collagen. In particular, the alterations in MMP-1 and MMP-13 protein expressions could have been responsible for the changed ratio of type I to type III collagen on the protein level. Nevertheless, as firstly shown in investigations by Bellon et al. in 1997, cultured fibroblasts in fascia transversalis from patients with inguinal hernia showed no differences in the expression of MMP-1, whereas the same author later detected a MMP-2 over expression in these patients [13,14]. These results on protein level appear to suggest that in comparison to MMP-1 and MMP-13, MMP-2 is an active part of degradation system of the extracellular matrix in hernia patients. Based on above facts, a hypothesis was generated that hernia is a local manifestation of a systemic disease which is manifested by increased expression of MMP-2. Thus a study was planned with research objectives to establish a causal association between hernia and MMP-2 and to test the hypothesis that hernia is a local manifestation of a systemic disorder rather than a mere local mechanical defect.

## Materials and Methods

A case control study was designed in which patients admitted in the department of general surgery of CSM Medical University (CSMMU), Lucknow, constituted the study and control group. In study group, patients who were operated for direct/indirect inguinal hernias (n = 30 each for direct and indirect hernia) in the department of general surgery, CSMMU, were included. Randomization was done according to Table of Random Number Method. Controls (n = 30) were age and sex matched patients who were operated for abdominal trauma in emergency OT, Trauma Centre, CSMMU. None of these patients had any type of abdominal wall hernia. Patients suffering from any type of connective tissue disorder and with chronically raised intra abdominal pressure, e.g. COPD, pregnancy, intra abdominal tumour etc., were excluded from the study.

### Sample collection and transportation

#### Serum samples

Blood samples were taken preoperatively. Serum was separated from blood after allowing it to stay in a test tube for about 30 minutes followed by centrifugation at 3000 rpm for 10 minutes. Serum was stored in eppendorf vials till further processing.

#### Tissue samples

In the study group, a section of about 1 x 1 cm of fascia transversalis tissue was taken while in the control group, section of rectus sheath was taken of same size. Tissue samples were kept in normal saline after washing with distilled water. Both serum and tissue samples were transported to the laboratory at National Botanical Research Institute, Lucknow, in ice cooled boxes within 2 hours of extraction. There these samples were kept at -70^o^C till commencement of the analysis.

Direct antigen coating ELISA (DAC-ELISA) test was done for detection of serum and tissue MMP-2 levels.

### Procedure

Serum samples were diluted in PBST buffer in different dilutions whereas tissue samples were crushed in liquid nitrogen in hepes buffer (pH 7.4) in 1 : 3 ratio (mg : μl) followed by determination of protein concentrations by dye binding procedure (Bradford, 1976) using bovine serum albumin (BSA) as standard protein. Determination of protein content was done by Bradford Assay, where 10μl of the protein crude extract was poured by pipette in the wells of micro titer plate and 200 μl of Bradford dye was added to each well. The absorbance was read at 595 nm. Finally, concentration of protein was determined by Bradford assay in μg/μl by following formula:

Amount of protein (μg/μl) = [(Sample mean O.D. - Blank O.D.) x S F]/Sample Volume

#### Determination of MMP-2 concentration by DAC-ELISA

The samples were diluted up to 3.0 μg/ml in bicarbonate buffer and 100 μl was loaded in duplicates with proper negative and positive control. Plates were incubated for 2 h at 37^o^C, then blocked with blocking buffer i.e. 5% NFDM (non fat dry milk) and incubated for 1 h at 37^o^C followed by incubation with primary antibody at 1 : 5000 dilution in blocking solution (primary antibody used was polyclonal rabbit anti human MMP-2-Sigma, USA) for 2 h. Plate was then blocked with 5% NFDM for 30 min at 37^o^C and the wells were incubated for 2 h with secondary antibody at 1 : 8000 dilutions in blocking solution (secondary antibody was goat anti-rabbit IgG conjugated to alkaline phosphatase). The plate was washed twice with PBST and twice with diethanolamine buffer. Substrate para nitrophenyl phosphate was added and the plate was kept at room temperature for 1 h. Then 50 μL 3 M sodium hydroxide was added to stop the reaction. Between each step of incubation, plates were washed with PBST. Absorbance at 405 nm was read by the interval of 15 min for 1 h using Spectra Max 340 PL spectrophotometer-Molecular devices, USA. Expression levels were quantified on a linear standard curve plotted with pure human MMP-2.

The concentration of MMP-2 is determined by the following formula:

Concentration of MMP-2 (ng/ml) = (Sample mean O.D. - Blank O.D.) x standard factor (where standard factor = 91.7)

## Results

**Figure 1:**
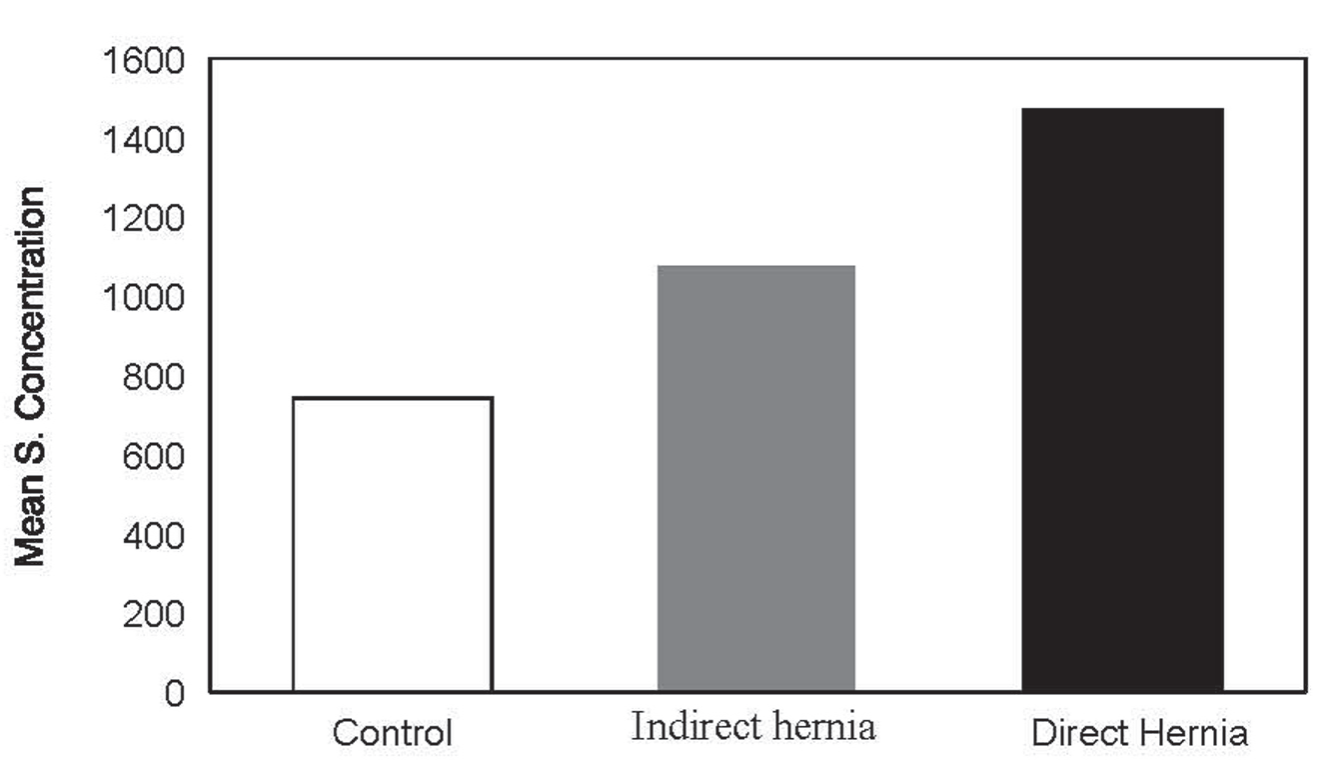
Figure 1. Serum concentration in hernia groups.

All the cases of direct and indirect hernia were males. Controls were aged between 22 to 57 years, and the mean age of control group subjects was 35.90 ± 10.67 years. The patients of indirect hernia were aged between 22 - 42 years with a mean age of 31.03 ± 5.60 years. The patients of direct hernia were aged between 33 - 60 years with a mean age of 49.33 ± 7.21 years. The mean serum concentration of MMP-2 in control group was 745.37 ± 30.05 ng/ml with a minimum of 667 and maximum of 803 ng/ml. The mean serum concentration in direct hernia group was 1473.37 ± 118.95 ng/ml with a range of 1244 - 1678 ng/ml while the mean value of serum MMP-2 concentration in indirect hernia group was 1076.07 ± 80.06 ng/ml with values ranging from 967 to 1247 ng/ml, thereby showing a statistically significant difference among groups (Table 1, Fig. 1). On the basis of above observations, the serum MMP-2 concentration could be shown as: Control < Indirect Hernia < Direct Hernia.

**Table 1:** Mean Serum Concentration of MMP-2 in Different Groups

	N	Mean	SD	Minimum	Maximum
Control	30	745.37	30.05	667	803
Indirect Hernia	30	1076.07	80.06	967	1247
Direct Hernia	30	1473.37	118.95	1244	1678

F = 557.145; p<0.001 (ANOVA)

### Analysis of tissue samples

We were not able to find any detectable amount of MMP-2 in tissue samples (neither in controls nor in test samples).

## Discussion

In a study conducted by J. Smigielski et al in 2007 [15], significant increase in serum MMP-2 levels of indirect, direct and recurrent inguinal hernia as compared to controls was found. There was also significant difference with respect to age. The levels were higher in subgroup of younger patients as compared to subgroup of older patients (highest levels were found in young patients with direct hernia). In our study the patients of direct hernia showed highest levels of serum MMP-2 but no significant difference was found with respect to age.

Juan M. Bellon et al [14] performed a cell culture of fibroblasts from fascia transversalis of young patients with direct inguinal hernia and observed significant expression of active MMP-2. These findings were confirmed by immunosorbent assay, immunoblotting, immunocytochemistry and zymography in the culture media. In our study, we directly used the tissue samples after crushing them in liquid nitrogen. Probably cell culture would have been beneficial for amplification of MMP-2 expression. Immunocytochemistry can be more sensitive but it requires long duration of time for sectioning, staining etc. We adopted a simple procedure which was easy to perform and required a shorter time span.

R. Rosch et al in 2005 [16] aimed to investigate the MMP-2 expression in patients with recurrent incisional hernias with and without mesh-materials. In primary fibroblast cultures obtained from skin scars of patients with and without recurrent incisional hernias, MMP-2 synthesis and gene expression were investigated. Furthermore, MMP-2 synthesis and gene expression of fibroblasts were compared after incubation with two different mesh materials: polypropylene and absorbable polyglactin filaments. Enzyme activity of MMP-2 was determined by semiquantitative zymography and mRNA synthesis by quantitative RT-PCR. Both MMP-2 enzyme activity and mRNA expression were similar in hernia and control fibroblasts in vitro. In control, fibroblasts mesh incubation did not significantly affect MMP-2 expression, whereas polypropylene mesh contact of fibroblasts from patients with recurrent incisional hernias led to a major decrease of MMP-2 activity and of mRNA expression. In the absence of biomaterials fibroblasts from recurrent hernia, patients have no alterations of their MMP-2 synthesis compared to control, whereas a specific response was found after biomaterial contact indicating the differences in fibroblast phenotype.

Based on international research and our own results, we found that increase in MMP-2 activity could be considered to play a significant role in the etiology of inguinal hernias. The increased activity may lead to the dysfunction of collagen fibers, which are responsible for forming fascial structures, and as a result, it can weaken their durability.

## Conclusions

Because there is statistically significant increase in serum MMP-2 levels in patients of indirect and direct hernia both as compared to controls, thus we can conclude that hernia is not a mere local defect but a local manifestation of a systemic disease. This is more apparent in patients of direct hernia group as mean serum concentration of serum MMP-2 is highest in these patients.
